# Patient-Reported Experiences and Satisfaction with Rural Outreach Clinics in New South Wales, Australia: A Cross-Sectional Study

**DOI:** 10.3390/healthcare10081391

**Published:** 2022-07-26

**Authors:** Md Irteja Islam, Claire O’Neill, Hibah Kolur, Sharif Bagnulo, Richard Colbran, Alexandra Martiniuk

**Affiliations:** 1 Sydney School of Public Health, Faculty of Medicine and Health, The University of Sydney, Camperdown, NSW 2006, Australia; alexandra.martiniuk@sydney.edu.au; 2 Centre for Health Research, Faculty of Health, Engineering and Sciences, The University of Southern Queensland, Darling Heights, QLD 4350, Australia; 3 NSW Rural Doctors Network, Suite 1, 53 Cleary St., Hamilton, NSW 2303, Australia; claire.oneill23@gmail.com (C.O.); sbagnulo@nswrdn.com.au (S.B.); rcolbran@nswrdn.com.au (R.C.); 4 Faculty of Arts and Science, Queen’s University, 99 University Ave, Kingston, ON K7L 3N6, Canada; h.kolur@queensu.ca; 5 Office of the Chief Scientist, The George Institute for Global Health, Level 5/1 King Street, Newtown, NSW 2042, Australia; 6 Dalla Lana School of Public Health, The University of Toronto, 155 College St. Room 500, Toronto, ON M5T 3M7, Canada

**Keywords:** Australia, experience, health services, outreach clinics, patient, rural, satisfaction

## Abstract

**Introduction:** Many studies have been conducted on how physicians view outreach health services, yet few have explored how rural patients view these services. This study aimed to examine the patient experience and satisfaction with outreach health services in rural NSW, Australia and the factors associated with satisfaction. **Methods:** A cross-sectional study was conducted among patients who visited outreach health services between December 2020 and February 2021 across rural and remote New South Wales, Australia. Data on patient satisfaction were collected using a validated questionnaire. Both bivariate (chi-squared test) and multivariate analyses (logistic regression) were performed to identify the factors associated with the outcome variable (patient satisfaction). **Results:** A total of 207 participants were included in the study. The mean age of respondents was 58.6 years, and 50.2% were men. Ninety-three percent of all participants were satisfied with the outreach health services. Respectful behaviours of the outreach healthcare practitioners were significantly associated with the higher patient satisfaction attending outreach clinics. **Conclusions:** The current study demonstrated a high level of patient satisfaction regarding outreach health services in rural and remote NSW, Australia. Further, our study findings showed the importance of collecting data about patient satisfaction to strengthen outreach service quality.

## 1. Introduction

Access to quality health care is important for all. Several frameworks and terms exist describing the categories and aim for health system reform toward improving the quality of healthcare. In recent years, patient-centred care has evolved as a key approach for improving health care [1,2], including in Australia [3]. The main objective of patient-centred care is to encourage partnerships between patients and healthcare practitioners, acknowledge patients’ preferences and beliefs, and allow flexibility in providing health care [2,4,5]. Moreover, the patient-centred care approach focuses on enhancing patient satisfaction [1,4,5].

Patient experience and satisfaction with their health care matters because well-being, recovering or living with a condition can be affected by the interactions a patient has with staff and the processes of health care [6]. Previous research demonstrates that good experiences of care are associated with good clinical and quality of life outcomes [6]. Patient satisfaction is measured as the difference between a patient’s expectations and the performance of the service against that standard [7,8,9]. This provides vital insights into the health care provided and assists in decisions toward improving healthcare policy and processes [10]. Satisfaction with health care encompasses both the technical and interpersonal aspects of care [9,11]. It is thought to consist of three domains: delivery of medical care, the treatments sought by patients, and the behaviour of the healthcare provider implicating compassionate care [10,11]. Contributing factors, such as age, gender, waiting time, communication, patient trust, and continuity of care are included within these three domains [12]. The previous literature has identified positive correlations between factors such as patient trust and patient satisfaction, and negative correlations between factors such as waiting time and patient satisfaction [11]. This has been supported by research on the impact of patient satisfaction on health, substantiating a cyclical relationship wherein higher patient satisfaction leads to higher patient adherence, improved clinical outcomes and less healthcare utilisation [13,14].

Over 44% of the global population inhabits rural areas, yet the centralization of healthcare services to urban cities has weakened the rural healthcare system [15]. Rural patients face greater challenges in accessing viable health services in the form of costly travel expenses, absenteeism from work and family, childcare expenses, and scarcity of required healthcare providers. People who live in rural areas carry a greater burden of comorbid and chronic diseases [15,16,17]. For example, previous research suggests rural people receive delayed treatment for brain and myocardial infarction [18]. Further, the lack of healthcare services in rural areas often prompts patients to seek alternative sources of medical care, such as telemedicine, outreach health clinics, role substitution with general practitioner proceduralists along with other practitioner disciplines and presenting for care through emergency rooms [19]. Orlando et al., found high satisfaction rates with telehealth models, providing enhanced communication and engagement between healthcare providers and patients [20]. Multiple factors, such as the ability to see nurses when needed, provision of pain relief, feeling comfortable and safe, and positive perceptions of nurses’ interest in patients were found to greatly enhance patient satisfaction with their health care [21].

In Australia, the healthcare system is a hybrid model in which citizens, permanent and temporary residents, as well as refugees can buy private insurance coverage in addition to the public insurance the government provides using tax-payer funds [22,23]. Furthermore, non-government organizations and state and federal governments provide healthcare services specifically for Australian Indigenous peoples [24]. Well-known barriers to primary care in Australia include low population density outside of major cities [25,26], low numbers of healthcare providers in rural and remote regions, and large distances to tertiary care. In the cities and regional centres, individuals can identify their own GP and typically can get an appointment on the same day, if not almost certainly in the same week of need. There are also home visiting GPs covered under the public system [27]. Outside of cities and regional health facilities, there may not be a GP within a reasonable distance, or existing GPs may not be taking new patients due to the full workload. This has resulted in different perceptions and experiences of the Australian health system between rural and urban populations, which were exacerbated by the COVID pandemic [28]. Outreach clinics have been employed as a key strategy toward increasing universal access to healthcare services, particularly in rural Australia, that benefits from nationally consistent policy. There is anecdotal evidence to suggest that regular medical specialist outreach services to remote and Aboriginal communities in Australia were established at the request of rural GPs as early as the 1950s and 1960s [29]. However, these service models were not sustainable in the longer term, as they generally relied on the altruism of medical specialists in the absence of other incentives.

Rural communities account for 28% of Australia’s population, out of which 62% are Indigenous Australians [25,30]. However, only 12% of medical specialists reside in rural areas [31,32]. Similarly, rural areas typically have fewer nurses and allied health professionals compared to urban areas [33,34]. On average, rural patients are 4.5 times more likely to travel over an hour to see a general practitioner than urban patients [24]. On the other hand, Indigenous people are more likely to avoid mainstream health services compared with non-Indigenous people in Australia, resulting in poor health outcomes [24,35]. To mitigate these health disparities, the Australian government introduced the Rural Health Outreach Fund in 2000. Through the program, health practitioners are reimbursed and provided subsidies for their involvement in outreach health clinics. Metropolitan healthcare workers account for over three-quarters of outreach providers due to their high specialist concentration [36,37,38]. Outreach clinics have been vital in improving clinical outcomes, reducing hospitalisations, reducing injury-related deaths, increasing life expectancy, and improving the mental health of rural populations [38]. In particular, specialist outreach embedded in holistic models involving collaboration with primary care, education and other services has been associated with improved health outcomes, more efficient care, and care adhering to guidelines and reductions in inpatient hospitalizations [39].

New South Wales (NSW) Health, responsible for delivery of publicly funded health care in the state, sees patient experience and satisfaction as important factors to understand and have implemented several initiatives aiming to gather data on patient experience (for instance, ‘Your Experience Matters’ in several NSW Local Health Districts (LHDs)) [40]. These ‘Your Experience Matters’ data are not collected during outreach services or available for outreach services, although outreach services are an important way for rural populations to receive their health services. To the best of our knowledge, no previous study has identified factors related to patients’ level of satisfaction with rural outreach health services in Australia, and major research has been conducted on physician points of view on the effectiveness of rural outreach health clinics. There is a considerable gap in the existing literature regarding the evaluation of patient experience and satisfaction with rural outreach clinics. Further, the importance of understanding patient experience has been underscored for Indigenous patients, yet while important, little quantitative data exist on Indigenous patients’ experience and satisfaction with the health care services they receive. Understanding patient experience and satisfaction with outreach health services is an important component in terms of understanding the quality of outreach health services.

Therefore, this study aimed to investigate patient-reported experience and satisfaction during the initial months and years of the COVID pandemic and the factors associated with patient satisfaction with outreach health services for those living in rural and remote settings where the service would otherwise be inaccessible in NSW, Australia. Increasing our knowledge regarding patients’ experiences and satisfaction with rural outreach clinics can assist in our understanding of gaps in health services and patient care and assist with curating future viable interventions aimed at improving access and quality of outreach clinical care in rural and remote settings.

## 2. Materials and Methods

### 2.1. Data Source

This study used data from a cross-sectional survey conducted within outreach clinics between December 2020 and February 2021 across New South Wales (NSW), Australia, endorsed by the NSW Rural Doctors Network (RDN). Each year, RDN routinely surveys patients visiting outreach clinics as part of their annual evaluation of services and for quality improvement. Briefly, RDN’s Outreach Program is funded by the Australian Government Department of Health and administered by RDN in NSW and the ACT. The overarching aim of RDN’s Outreach Program is to increase the types of services available in rural, remote, and Aboriginal communities and contribute to improving health outcomes in these communities. Outreach services included the following programs: Rural Health Outreach Fund, Medical Outreach Indigenous Chronic Disease Program, Healthy Ears, Better Hearing, Better Listening Program, Ear and Eye Surgical Support Service, and the Visiting Optometrists’ Scheme [41].

The RDN Outreach programs on average report about 200,000 patient occasions of service each year. This questionnaire-based survey was routinely conducted in a convenience sample of patients who attended the outreach clinics either in-person or via telehealth. Only patients who voluntarily agreed to participate in the study were included. All agreed participants were given a survey questionnaire with a written informed consent form after their medical consultation for self-completion at the reception for in-person visiting patients, while telehealth consultation patients were provided with an online link to the survey via text message after consultation. If a patient was under the age of 18, a parent or guardian was requested to complete the survey, if possible. All respondents were assured of complete confidentiality and anonymity. In most cases, patients understood the statements, found them straightforward to fill out, and anonymously dropped them in the sealed box within 15 min of receiving them.

The RDN programs fund approximately 1200 services each year. Each service may be funded to conduct from 1 to approximately 50 visits (most of the time visits = clinics) in a 12-month period. Initially, RDN randomly selected 100 outreach services across NSW in which to conduct surveys; however, only 32 of the selected services participated. This is said to be because surveys were distributed during the COVID-19 pandemic. It is also likely that some of the services selected to survey did not have a clinic date planned for the survey period, or the type of service may not have been appropriate to survey. A total of 229 survey forms were returned compared to 389 the previous year. Patients who provided incomplete responses or refused to answer (*n* = 22) were excluded from the analysis, and therefore, a total of 207 samples were included in the analysis. 

#### Measures

Based on published scientific evidence [8,9,42], a structured questionnaire (Supplementary File S1) was designed; however, the survey team modified the questions to make them more specific for rural Australian outreach clinic patients. The questionnaire gathered data on the sociodemographic characteristics of the participants, followed by questions exploring the type, accessibility, health professional-patient relationship, cost, and convenience of outreach services (experiences). The questionnaire also measured the overall satisfaction of the patients with the outreach health care received and allowed respondents to make suggestions to improve the quality of services. The reliability of the questionnaire was measured by the Cronbach alpha coefficient (α = 0.58). 

### 2.2. Preparing the Data for Analyses

Patients’ overall satisfaction with outreach health services was selected as an outcome variable. Patients’ satisfaction was measured with the following question: ‘Overall, how satisfied were you with the service you received today in the outreach clinic?’, rated on a five-point Likert scale (very dissatisfied, dissatisfied, neither dissatisfied nor satisfied, satisfied, very satisfied). For the descriptive analyses, we created a dichotomized variable, ‘patient satisfaction’, from the responses. Patients who responded ‘satisfied’ or ‘very satisfied’ were classified as ‘satisfied’ (coded as 1), while those who answered, ‘very dissatisfied’, ‘dissatisfied’, or ‘neither dissatisfied nor satisfied’ were classified as ‘dissatisfied’ (coded as 0).

The following variables were used as potential covariates in the analyses: age (children, adults, and older adults), gender (male and female), country of birth (overseas and Australia), Indigeneity (Indigenous and Non-Indigenous), type of health professionals visited (General Practitioner (GP), Specialists, and others including Aboriginal Health Worker, Nurse, Therapist, Nutritionist), mode of consultation (telehealth/online and in-person), type of transport used (no transport, public and private), travel time to reach outreach clinics (<20 min and ≥20 min), and frequency of visits (once and twice/more). In addition, we evaluated patients’ experiences related to the patient’s last outreach clinic visit using the following question: ‘Did the Health Professional treat you respectfully?’. For the analyses, a binary variable was created as ‘Behaviour of Health Professional’ from the responses, coded as 0 for ‘Not respectful’ and coded as 1 for ‘Respectful’.

Survey weight variables were created using the total number of rural populations in NSW.

### 2.3. Statistical Analyses

Initially, sample (*n* = 207) characteristics were outlined by descriptive statistics in terms of frequency (*n*) and percentages (%) with a 95% confidence interval (CI) for all potential sociodemographic variables. Bivariate analyses were then conducted to examine the explanatory variables and their distributions over the primary outcome variable (overall patient satisfaction with the outreach services). The Pearson’s Chi-squared tests [43] signified the strength of the bivariate relationships. Later, logistic regression models were employed to identify the variables associated with overall patients’ satisfaction with outreach services. Statistically significant covariates (*p* < 0.05) in bivariate analyses were only included in the adjusted logistic model. The results of the logistic regression analyses were presented as adjusted odds ratios (aOR) with their corresponding 95% CI and *p*-values.

Finally, the assumptions of logistic regression were evaluated. For example, McKelvey and Zavoina’s R^2^ [44] and goodness-of-fit test [45] for model performance, the Link test [46] for model specification, and the variance inflation factor (VIF) test [47] for detecting multicollinearity among predictors in the model were utilized. All the analyses were conducted using Stata/SE 14.1 (StataCorp, College Station, TX, USA) and the ‘SVY’ command was used to account for survey design and survey weights [48].

### 2.4. Ethical Information

In alignment with the National Statement on Ethical Conduct in Human Research, this study used a routinely collected and completely unidentifiable dataset. We accessed, analysed and presented the results from the data in a non-identifiable form. This type of secondary data fits with the University of Sydney Research Ethics Board’s Outcome A and does not require ethical committee approval (Supplementary File S2).

## 3. Results

The sample comprised 207 patients who attended the outreach clinics delivered through the Rural Doctors Network, local partner agencies and health practitioners across NSW, Australia. Table 1 summarizes the characteristics of the respondents. The mean age of the sample was 58.6 years (SD = ±20.5), 104 (50.2%) were males, and 103 (49.8%) were females. Most participants were from Australia (187, 90.3%) and roughly two-thirds were not Indigenous people (132, 63.8%). Indigenous health services may have been more impacted by COVID-19 which may have impacted the respondent proportions for this survey.

It was found that the majority of the participants (81.6%) visited the medical outreach health professionals, and more than 90% of patients preferred in-person consultation. About 71% of the participants used their own vehicle to reach the clinic for consultation, and 75.4% had at least 2 visits in the past 12 months due to illness. Table 1 also shows that nearly 89% of patients perceived respectful behaviour from their healthcare professionals.

**Figure 1:**
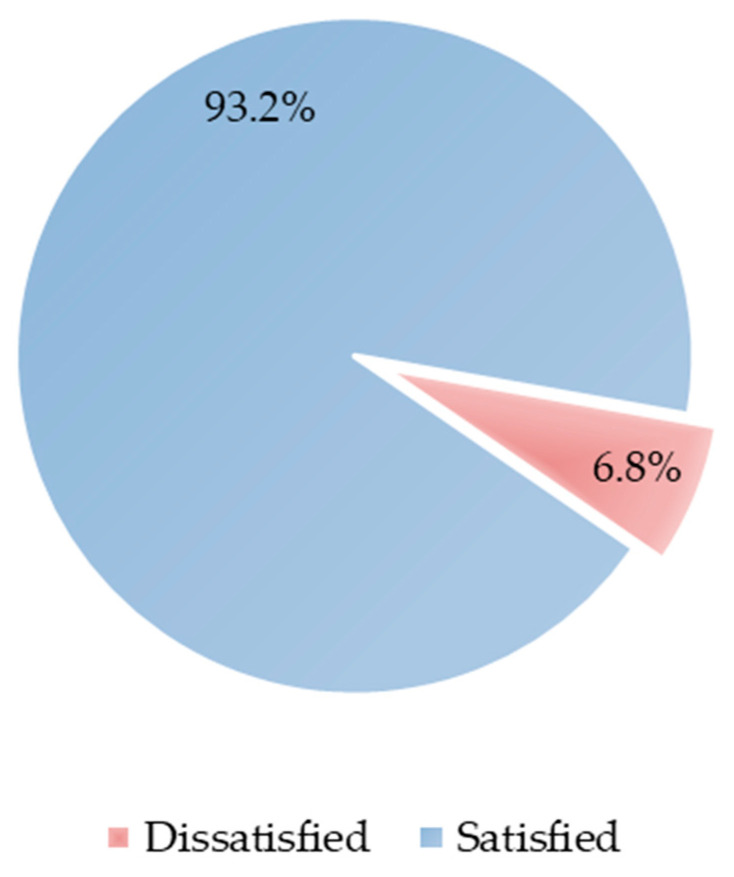
Patients’ Satisfaction vs Dissatisfaction with outreach health services.

The rates of overall satisfaction of patients with the outreach clinics are shown in Figure 1. Out of a total of 207 participants, most of the participants (193, 93%) were satisfied with the service received at the outreach clinics.

Further, the findings from the bivariate associations between potential covariates and patients’ satisfaction with outreach clinics are portrayed in Table 2. The comparisons between the participants’ satisfaction and dissatisfaction with the outreach services showed that satisfaction significantly differs by type of transport (*p* < 0.05), frequency of visits (*p* < 0.05) and the experience of being treated by health professionals with respect (*p* < 0.001).

The results of the binary logistic regression model are depicted in Table 3. It was observed that the respectful behaviour of outreach health professionals was significantly associated (*p* < 0.01) with higher patient satisfaction compared to those who were not respectful to their patients, while, unfortunately, the analysis of developed indices presented in Table 3 confirmed that the transportation used by the patients to reach outreach clinics and the frequency of visits by the patient to the outreach clinics were not significantly associated with the overall satisfaction compared to their counterparts.

Furthermore, Table 3 depicts the results obtained from regression diagnostic tests to safeguard precise estimation. For instance, the VIF with a mean of 1.05 (maximum 1.07) confirmed no evidence of multicollinearity among the predictor variables. McKelvey and Zavoina’s R^2^ value was less than 1.0, and the goodness-of-fit test statistics demonstrated that there was no significant discrepancy between the model and the observed data (*p* > 0.05), indicating that the model used was correctly fitted. Finally, the Link test (*p* < 0.05) validated that the model was appropriately specified.

## 4. Discussion

Studying patients’ experiences and satisfaction with health care increases our understanding of the socio-psychological aspects of patient-health professional interactions but also supports the ongoing development of patient-focused paradigms of medical and allied health services [49,50,51,52]. This study examined patients’ experiences and satisfaction and provided empirical evidence quantifying the factors associated with patients’ satisfaction with outreach health services in rural and remote areas of NSW, Australia. Patient satisfaction with health services is an important factor because it is associated with adherence to care recommendations and therefore health outcomes. This study has identified the respectful behaviour of health professionals as an important determinant of patients’ satisfaction in attending rural outreach clinics given that 62% of Indigenous Australians live in rural areas, and a culturally safe environment is essential for engaging Indigenous people in health services. The findings of this paper are essential and novel in drawing connections between patient experience and satisfaction with rural outreach services during initial months of COVID focused on close relationships with local providers facilitated by RDN.

This study revealed important differences in satisfaction for Indigenous people. Higher patient satisfaction scores were found among Indigenous patients. This finding was expected from the data because the majority of the services funded through the RDN outreach program are designed and delivered by Aboriginal health organisations, which are thought to address cultural safety needs. According to the Organisation for Economic Co-Operation and Development (OECD), developing and implementing a patient-centred, culturally safe approach is vital for improving the Australian Indigenous health at all levels and components of the healthcare system [53]. Evidence also suggests higher satisfaction with health services among Indigenous patients can be achieved by adequately involving Indigenous health professionals in a culturally safe environment. While also improving Indigenous and non-Indigenous health workers’ knowledge of culturally specific signs of sickness and ways of providing culturally sensitive care to Indigenous patients [54,55,56].

It is notable, that the highest overall satisfaction was reported by patients who were visiting physicians (GPs and Specialists) at the outreach clinics as compared to non-medical outreach health professionals. This may be because outreach medical services in Australia are typically provided by specialist doctors who periodically visit the same rural community without any health service fees charged to the patient [32,38]. 

Importantly, the results also revealed that patients were more satisfied with outreach services when they were treated with respect. This finding was congruent with those of other authors’ published research, confirming that positive behavioural communication with the patient can have an impact on overall satisfaction [8,9]. Subsequent data depicted that 89% of patients found the outreach health professional respectful, further mirroring previous data collected on this factor in the nationally representative Australian Bureau of Statistics Patient Experience Survey (PES) [57]. Further examples can be found in studies conducted by Clever et al. [58] and Biglu et al. [59], which found that positive communication behaviour from physicians increases patients’ satisfaction with the services. The level of satisfaction of patients increases with the service they receive [6,59]. This finding further substantiates that the patient-doctor relationship is key to quality health care [60,61]. Another study reported that positive and culturally competent healthcare services to Indigenous patients helps to build trust among Indigenous communities and increases positive health outcomes [24,62]. Strong patient-doctor relationships also improve patients’ knowledge and confidence about health conditions and increases active involvement in making their treatment plans and better prognosis [63]. Previous research also suggests that healthcare professionals with strong communication skills increase patients’ satisfaction, which has an impact on their overall rating of health services [64,65]. For example, health professionals’ ability to explain medical terms in layman’s terms or to communicate in a patient’s native language can help patients comprehend their prognosis, resulting in greater patient satisfaction [66]. The findings presented in this study provide important information for policymakers, managers, and health service providers who aim to improve the quality of outreach health services to rural communities, including Indigenous people [60,61,63]. It is known that positive patient experiences are associated with clinical effectiveness and improved patient safety [67]. Gathering data on patient experiences and satisfaction is an important adjunct to understanding the safety and quality of health services.

There are some limitations to this study. First, the study uses a cross-sectional study design, so it was not possible to attribute temporal causality between any of the variables. Other limitations include the possibility that selection and response biases have impacted the study findings as the study needed to use a convenience sample, a non-probability form of sampling technique. Moreover, our study findings may not be generalizable to the general population due to the convenience and small sample. Future research using qualitative study and/or longitudinal study designs may build upon this new knowledge by collecting data on other variables, which may impact patient experience and satisfaction, such as waiting times, waiting room conditions, time spent by the health professionals with the patient, alternatives to the care, the severity of the condition, frequency of usual health care and frequency of outreach health service availability. Lastly, it is worthwhile to mention that the reliability of the used questionnaire was low and was not validated. Further research is recommended in outreach settings to better understand the correlation between patients’ experiences and satisfaction with their health care and clinical outcomes.

## 5. Conclusions

Overall, patients’ satisfaction with the services is a direct result of improved communication and trust, which leads to better treatment adherence and health outcomes. This study has shown that currently most of the patients who received health care via an outreach clinic were fully satisfied. Respectful behaviour of health professionals was significantly associated with higher patients’ satisfaction with attending outreach clinics. Moreover, the current study demonstrates the potential to gather and use data on patients’ experience and satisfaction as additional measures to understand the quality of outreach health services in rural areas of Australia, including Indigenous communities.

## Figures and Tables

**Table 1:** Sample characteristics (*n* = 207).

	*n* (%)	95% CI
Age (Mean = 58.6, SD = ±20.50)		
Adults (18–64 years)	106 (51.2)	0.45–0.58
Children (≤17 years)	9 (4.4)	0.02–0.08
Older adult (≥65 years)	92 (44.4)	0.38–0.51
Gender		
Male	104 (50.2)	0.43–0.57
Female	103 (49.8)	0.42–0.56
Country of birth		
Australia	187 (90.3)	0.85–0.93
Overseas	20 (9.7)	0.06–0.14
Indigeneity		
Non-Indigenous	132 (63.8)	0.56–0.71
Indigenous	75 (36.2)	0.29–0.43
Health professionals visited ^1^		
Medical	169 (81.6)	0.75–0.86
Non-Medical	38 (18.4)	0.14–0.24
Mode of consultation		
In-person	189 (91.3)	0.86–0.95
Telephone or Online	18 (8.7)	0.05–0.13
Type of transport		
Private	148 (71.5)	0.65–0.78
Public	35 (16.9)	0.12–0.22
No transport or Walk	24 (11.6)	0.07–0.16
Travel time		
<20 min	117 (56.5)	0.49–0.63
≥20 min	90 (43.5)	0.36–0.50
Frequency of visits ^2^		
Twice or more	156 (75.4)	0.69–0.8 1
Once	51 (24.6)	0.19–0.31
Behaviour of Health Professionals		
Not respectful	23 (11.1)	0.08–0.16
Respectful	184 (88.9)	0.83–0.92

^1^ Health professional visited by the patient: Medical—GP (General practitioner) and Specialists, and Non-medical (i.e., Aboriginal health worker, Nutritionist, Psychologists, Social therapist, and Nurse), ^2^ Frequency of visits—Number of times patients visited the outreach health services in the last 12 months due to illness. SD, standard deviation. CI, confidence interval.

**Table 2:** Bivariate association between sociodemographic factors and patients’ level of satisfaction.

	Dissatisfied	Satisfied	Pearson χ^2^ (*p*-Value)
	*n* (%)	*n* (%)	
Age (Mean = 58.6, SD = ±20.50)			4.21 (0.121)
Adults (18–64 years)	5 (4.7)	101 (95.3)	
Children (≤17 years)	2 (22.2)	7 (77.8)	
Older adult (≥65 years)	7 (7.61)	85 (92.4)	
Gender			0.32 (0.567)
Male	6 (5.8)	98 (94.2)	
Female	8 (7.8)	95 (92.2)	
Country of birth			0.11 (0.741)
Australia	13 (6.9)	174 (93.1)	
Overseas	1 (5.0)	19 (95.0)	
Indigeneity			0.38 (0.537)
Non-Indigenous	10 (7.6)	122 (92.4)	
Indigenous	4 (5.3)	71 (94.7)	
Health professionals visited			0.09 (0.759)
Medical	11 (6.5)	158 (93.5)	
Non-Medical	3 (7.9)	35 (92.1)	
Mode of consultation			3.06 (0.080)
In-person	11 (5.8)	178 (94.2)	
Telephone or Online	3 (16.7)	15 (83.3)	
Type of transport			7.79 (0.020 *)
Private	6 (4.1)	142 (95.9)	
Public	6 (17.1)	29 (82.9)	
No transport or Walk	2 (8.3)	22 (91.7)	
Travel time			0.36 (0.544)
<20 min	9 (7.7)	108 (92.3)	
≥20 min	5 (5.6)	85 (94.4)	
Frequency of visits			5.20 (0.023 *)
Twice or more	7 (4.5)	149 (95.5)	
Once	7 (13.7)	44 (86.3)	
Behaviour of Health Professional			69.18 (<0.001 ***)
Not respectful	11 (47.8)	12 (52.2)	
Respectful	3 (1.6)	181 (98.4)	

Level of significance: * *p* < 0.05, *** *p* < 0.001. Row percentages (%) presented.

**Table 3:** Determinants of patient satisfaction—Logistic model.

	aOR	95% CI	VIF
Vehicle used (Ref. Private)			1.07
Public	0.11 ***	0.08–0.17	
No transport or Walk	0.12	0.00–15.95	
Frequency of visits (Ref. Twice or more)			1.07
Once	0.25	0.03–2.10	
Behaviour of Health professionals (Ref. Not respectful)			1.00
Respectful	159.41 **	28.78–883.25	
Model statistics			
McKelvey and Zavoina’s R^2^	0.578		
Goodness-of-fit test	7.18		
Link test	7.68 **		
Mean VIF	1.05		

Level of significance: *** *p* < 0.001, ** *p* < 0.01. aOR, Adjusted odds ratio. CI, confidence interval. VIF, variance inflation factor.

## Data Availability

The data presented in this study are available on request from the Rural Doctors Network Survey team. The data are not publicly available as it is routinely collected data for patients visiting outreach clinics as part of their annual evaluation of services and for quality improvement.

## Supplementary Materials

The following supporting information can be downloaded at: https://www.mdpi.com/article/10.3390/healthcare10081391/s1, File S1: NSW Rural Doctors Network (RDN) Outreach Patient Survey; File S2: Using Existing Data Collections in Research: Guidelines for Researchers
