# Supplementary material for: Patient-Reported Experiences and Satisfaction with Rural Outreach Clinics in New South Wales, Australia: A Cross-Sectional Study
Source: Healthcare (Basel). 2022 Jul 26;10(8):1391. doi: 10.3390/healthcare10081391 (PMC9332042; doi:10.3390/healthcare10081391)
Supplement: Supplementary file 1 [file healthcare-10-01391-s001.zip › healthcare-1773019-file S1.pdf]

NSW Rural Doctors Network (RDN) Outreach Patient Survey

The purpose of this survey is to help RDN understand how this service impacts your community and support high quality healthcare. By completing this survey, you consent to your deidentified responses being used to inform a report, benchmarking and/or research that may be published. It is not a requirement to complete this survey and no personal information will be collected, unless you choose to provide this after completing this survey. Information collected through this survey will be treated under RDN's privacy policy which can be found on our website at https://www.nswrdn.com.au/

If you have any questions about this survey, please contact RDN's Outreach Team on (02) 8337 8100.

About you:

Q1. Are you filling this survey out as a patient, carer or parent?

☐ Patient ☐ Carer ☐ Parent

Note: If you are a carer or parent, please complete these questions on the patient's behalf.

Q2. What is your current gender identity?

☐ Male ☐ Female ☐ Transgender
☐ Do not identify as female, male or transgender ☐ Prefer not to say

Q3. How old are you? _______ years.

Q4. Are you of Aboriginal or Torres Strait Islander origin?

☐ No ☐ Yes, Aboriginal ☐ Yes, Torres Strait Islander
☐ Yes, both Aboriginal and Torres Strait Islander ☐ Prefer not to answer

Q5. In which country were you born? ____

Q6. What language do you mostly speak at home? ____________________________________

Your appointment and health practitioner:

Q7. What type of health professional did you see today? (e.g. psychiatrist, endocrinologist, dietitian, podiatrist, chronic disease nurse)

____

Q8. Did you attend the consultation with the health professional in person, or via telehealth?

☐ In person (skip to question 11) ☐ Via telephone (audio only) ☐ Via video conference (audio and visual)
☐ Other ____

NSW RURAL DOCTORS NETWORK

Q9. If you attended this appointment via telehealth (telephone or video), did you join from your local health facility (i.e. hospital, GP practice, community health facility) or a private location (such as your home)?

□ Health facility □ Private location □ Other: ______________________________

Q10. Regarding the use of telehealth, tick all that apply:

☐ I have reliable access to telehealth equipment (i.e phone/tablet/computer)
☐ The phone/internet connection is reliable
☐ I am comfortable using telehealth equipment (i.e phone/tablet/computer)

Q11. In what town did this appointment take place? (if the appointment occurred via telehealth please state the town you were in for the appointment) ____

Q12. How did you travel to your appointment today?

☐ Private car (either driving yourself or being driven by a family member or friend)
☐ Community transport ☐ Public transport ☐ Walked ☐ Other (please specify)
□ No travel required

Q13. How long did it take you to travel to this appointment (one way)?

________ hours / or _______minutes or □ No travel required

Q14. How long did you have to wait for your appointment once you received a referral to this health professional?

☐ Within 1 week ☐ Within 1 month ☐ Within 1 – 3 months
☐ Up to 6 months ☐ More than 6 months

Q15. How many times have you attended appointments with this health professional in the last year?

☐ This is my first appointment ☐ Once ☐ Twice ☐ Three times
☐ More than three times

Q16. Did the health professional you saw refer you to another health professional?

☐ No
☐ Yes – please specify what health professional you were referred to ______________________________
- Do you have access to this health professional in your community? □ Yes □ No

NSW RURAL DOCTORS NETWORK

Q17. Were you charged a fee to access this appointment?

☐ Yes ☐ No

Please provide any comments you have about fees for this service;

____

Q18. Overall, how would you rate your satisfaction with this appointment?

☐ Very Satisfied ☐ Satisfied ☐ Dissatisfied ☐ Very Dissatisfied

Please explain the reason for your answer:

____

____

Q19. I felt the health professional was respectful of my cultural background (such as culture, beliefs, religion, race)

☐ Strongly agree ☐ Agree ☐ Disagree ☐ Strongly disagree

Additional comments: ____

Q20. Do you have any comments or suggestions about how to improve this service? ________________

____

____

Q21. What computer or mobile devices do you currently use in your daily life? (please tick all that apply)

☐ None ☐ Smart phone (e.g. iPhone or Android) ☐ Tablet (e.g. iPad or Android tablets)

☐ Laptop or Desktop ☐ Other (please specify) ________________________________

Q22. If this service was not available, how would you access this type of appointment?

I would...

- ☐ Access a bulk-billed or public health professional that is available in my town
- ☐ Pay a fee to access a health professional that is available in my town
- ☐ Use a video conference appointment that I can access from my town
- ☐ Travel to another town or city
- ☐ It is unlikely I would access this type of appointment
- ☐ I am not sure

About your health:

This section of the survey is taken from the Dartmouth COOP Functional Assessment Charts/WONCA (Copyright Dartmouth COOP group (Nelson et al. 1987) Dartmouth COOP Project 1995, WONCA (Scholten & Van Weel, 1992)

Please circle your response for the below questions:

Q27. How would you rate your overall health now compared to last time you saw this health professional?

Or… ☐ please tick if this is your first appointment

Much better	A little better	About the same	A little worse	Much worse
▲▲ ++	▲ +	◀▶ =	▼ −	▼▼ −−

Q28. During the past 2 weeks… How would you rate your health in general?

Excellent	Very Good	Good	Fair	Poor
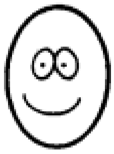	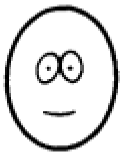	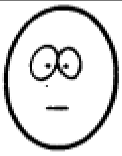	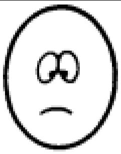	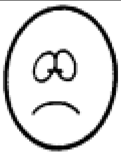

Thank you

Thank you for taking the time to complete this survey, your feedback is very important to us.

If you wish to be contacted regarding this survey please provide your contact details below:

Name: ___________________________

Phone Number: __________________

The information you provided in the above survey is anonymous; No personal information will be given to another organisation. If you would like to discuss your experience in more detail, please speak to a member of staff at the health facility hosting this service.
If you have any questions about this survey or the Outreach program, please contact Outreach@nswrdn.com.au
